# Non-Competitive Binding of Isatuximab and Daratumumab to CD38: Implications for Targeted Therapy in Multiple Myeloma

**DOI:** 10.3390/pharmaceutics17101278

**Published:** 2025-09-30

**Authors:** Rubén Osuna-Gómez, Jordi López-Pardo, Maria Mulet, Josep Nomdedéu, Elisabet Cantó, Rosa Ortin, Ángela Sánchez Cayuela, Ma Àngels Ortiz, Albert Guinart-Cuadra, Silvia Vidal

**Affiliations:** 1 Inflammatory Diseases, Institut de Recerca Sant Pau (IR Sant Pau), 08041 Barcelona, Spain; mmulet@santpau.cat (M.M.); ecanto@santpau.cat (E.C.); mortiz@santpau.cat (M.À.O.); aguinart@santpau.cat (A.G.-C.); 2 Haematology Department, Hospital de la Santa Creu i Sant Pau, 08041 Barcelona, Spain; jlopezpar@santpau.cat (J.L.-P.); jnomdedeu@santpau.cat (J.N.); rortin@santpau.cat (R.O.); asanchezcay@santpau.cat (Á.S.C.)

**Keywords:** multiple myeloma, monoclonal antibodies, CD38, cytotoxicity

## Abstract

**Background/Objectives**: CD38-targeting monoclonal antibodies isatuximab and daratumumab have revolutionized multiple myeloma (MM) treatment, but a deeper understanding of their distinct mechanisms is crucial for therapeutic optimization. **Methods**: We used flow cytometry to assess isatuximab and daratumumab binding competition in MM cell lines and patient-derived bone marrow cells. The dynamics of CD38 expression were evaluated at different time points before and after antibody-mediated removal. The effects of IMiDs (pomalidomide, lenalidomide) on CD38 expression and isatuximab-induced apoptosis, either alone or in combination with IMiDs, were also examined. Moreover, MM cell migration was assessed through CXCR4-mediated assays, and cell adhesion was evaluated via CD49d-dependent assays. **Results**: Isatuximab and daratumumab did not compete for CD38 binding, confirming distinct epitope recognition. Following depletion with either antibody, CD38 expression on the MM cell surface began to recover within 2 h, suggesting a dynamic regulation of CD38 availability. While daratumumab lacked direct apoptosis, isatuximab induced significant direct cell death. Pomalidomide enhanced isatuximab-induced apoptosis by increasing CD38 expression, whereas lenalidomide had no significant effect. Additionally, both antibodies effectively inhibited MM cell migration and significantly reduced cell adhesion. **Conclusions**: Their non-competitive binding and shared impact on cell dynamics suggest opportunities for optimizing treatment strategies through combinatorial or sequential approaches in MM therapy.

## 1. Introduction

Multiple myeloma (MM) is the second most common hematologic malignancy worldwide, and, despite advancements in treatment, it remains incurable [1,2]. MM is characterized by the clonal expansion and accumulation of malignant plasma cells in the bone marrow (BM), which disrupts normal hematopoiesis and results in clinical complications such as bone lesions, anemia, renal impairment, and hypercalcemia [3].

The high and uniform expression of CD38 on MM cells, compared with lower expression on most normal tissues, makes it an attractive therapeutic target [4,5]. CD38, a transmembrane glycoprotein, plays a pivotal role in various biological processes, including enzymatic activity, signal transduction, immune regulation, and cellular metabolism [4,6,7]. The extensive extracellular domain of CD38 allows it to engage in both frontal and lateral interactions with other receptors, modulating several immune functions [8,9]. Its biological roles extend beyond proliferation to the regulation of the tumor microenvironment, immune evasion, and metabolic adaptation [10,11,12,13,14]. Recent studies have highlighted CD38’s role in adhesion and tissue invasion [15]. CD38 cooperates with CXCR4. This is a chemokine receptor whose principal ligand is CXCL12, a key regulator of cell migration and homing to the BM [16]. CD38 can enhance adhesion mediated by CD49d, the α4 integrin subunit of VLA-4 (Very Late Antigen-4), which regulates leukocyte binding to vascular cell adhesion molecule-1 (VCAM-1) [17].

Targeting CD38 with monoclonal antibodies has revolutionized MM treatment, emphasizing the importance of understanding CD38’s complex biology to enhance therapeutic strategies [10]. Currently, two CD38-directed monoclonal antibodies are approved: daratumumab (human IgG1κ) and isatuximab (humanized IgG1κ). Both have shown efficacy as monotherapy and in combination with immunomodulatory drugs (IMiDs) and proteasome inhibitors (PIs) in newly diagnosed and relapsed/refractory MM (RRMM) [18,19,20]. Recent real-world studies have demonstrated that isatuximab, in combination with pomalidomide and dexamethasone, can induce meaningful responses in RRMM patients to daratumumab, highlighting its potential to overcome resistance through mechanisms distinct from daratumumab [21,22,23].

However, isatuximab and daratumumab bind to adjacent epitopes which may confer different activities [24,25]. To varying degrees, both antibodies trigger a variety of mechanisms, including cellular cytotoxicity and apoptosis induction [24,25]. In peripheral blood and BM, both daratumumab and isatuximab modulate T-cell immunity by decreasing regulatory T cells (Tregs) and enhancing effector T cell response [26,27]. Daratumumab induces broader changes, including an expansion of helper and cytotoxic T cells and increased T-cell clonality, whereas isatuximab preferentially targets Tregs, reducing their immunosuppressive profile and restoring conventional T-cell function [28,29]. Daratumumab-induced antibody-dependent cellular cytotoxicity (ADCC) can be augmented by lenalidomide and bortezomib, while isatuximab and pomalidomide demonstrate synergistic ADCC induction both in vitro and in vivo [20,30]. Notably, isatuximab is the only CD38 antibody capable of directly inducing apoptosis via caspase-dependent pathways in MM cells, independent of Fc fragment binding to Fc receptors [8,10,30]. In contrast, daratumumab requires secondary cross-linking or effector cells to initiate apoptosis [24,31].

Although isatuximab and daratumumab target different CD38 epitopes and have partially distinct functions, it remains unclear whether they compete for binding and how their functional differences are fully manifested. We hypothesized that characterizing these aspects would provide key insights with clinical relevance, helping to guide optimal treatment strategies with these antibodies in MM. Furthermore, we proposed that differences in their capacity to induce direct apoptosis and to modulate migration and adhesion may have implications for overcoming resistance and improving patient outcomes. To address these questions, we (i) analyzed the competition between daratumumab and isatuximab for CD38 binding using MM cell lines and BM plasma cells, (ii) determined the kinetics of CD38 surface recovery after antibody binding and removal, and (iii) compared the functional effects of both antibodies on direct apoptosis induction, cell migration, and adhesion. Together, these studies aim to generate mechanistic insights that may help optimize the use of CD38-targeted antibodies in clinical practice.

## 2. Materials and Methods

### 2.1. Cell Lines, Reagents and BM Samples

MM cell lines (MM.1S and MOLP-8) were purchased from American Type Culture Collection (ATCC, Manassas, VA, USA) and cultured in complete RPMI 1640 medium supplemented with 10% fetal calf serum (Biological Industries, Kibbutz Beit Haemek, Israel), 1X glutamine (Biological Industries), and 1X penicillin with streptomycin (Biological Industries) at 37 °C in a humidified 5% CO_2_ incubator. Daratumumab was obtained from Janssen Pharmaceuticals. Isatuximab was provided by Sanofi and was labeled with biotin using the Mix-n-Stain Kit (Sigma-Aldrich, St. Louis, MO, USA) according to the manufacturer’s instructions. Isatuximab was provided by Sanofi and was labeled with biotin using the Mix-n-Stain Kit (Sigma-Aldrich, St. Louis, MO, USA) according to the manufacturer’s instructions. Briefly, 100 µL of isatuximab at 1 mg/mL in 0.1 M sodium bicarbonate buffer (pH 8) was added to the vial of biotin and gently inverted. The reaction mixture was incubated for 1 h at room temperature (RT) in the dark. The unbound dye was removed using a purification column. Pomalidomide and lenalidomide were purchased from Celgene (Summit, NJ, USA).

This study was conducted at Hospital de la Santa Creu i Sant Pau between 2023 and 2024. The study population included five newly referred patients to the MM clinic for whom BM aspiration was performed during this period. All patients had a confirmed MM diagnosis based on current guidelines [32]. Written informed consent was obtained from all participants, and the study was approved by the Institutional Ethics Committee of Hospital de la Santa Creu i Sant Pau. All experiments with MM cell lines were performed in five independent assays.

### 2.2. Assessment of Daratumumab and Isatuximab Binding

MM.1S and MOLP-8 cell lines (1 × 10^6^ cells in 5-mL tubes) were incubated with 10 µg/mL of daratumumab and/or 10 µg/mL of biotinylated isatuximab at 37 °C for 30 min. After washing with PBS, cells were treated with streptavidin-APC (Miltenyi Biotec, Bergisch Gladbach, Germany) and/or anti-IgG1-BV421 (BD Bioscience, San Jose, CA, USA) for 15 min at RT. Then, cells were washed and stained with anti-CD38-PE-Vio770 (clone REA671; Miltenyi Biotec) for 15 min at RT. Subsequently, cells were centrifuged at 300× *g* 5 min and resuspended with 200 µL of PBS to be acquired by flow cytometry.

For BM cells from MM patients, 1 × 10^6^ cells in 5-mL tubes were lysed using red blood cell (RBC) lysis buffer (BioLegend, San Diego, CA, USA) at 37 °C for 10 min. After washing with PBS, cells were incubated with 10 µg/mL of daratumumab and/or biotinylated isatuximab at 37 °C for 30 min. Then, cells were washed and incubated with streptavidin-APC (Miltenyi Biotec) and/or anti-IgG1-BV421 (BD Bioscience), anti-CD38-PE-Vio770 (clone REA671; Miltenyi Biotec), and anti-CD138-PE (Miltenyi Biotec) for 15 min at RT. Afterwards, cells were centrifuged at 300× *g* 5 min and resuspended with 200 µL of PBS to be acquired by flow cytometry.

For BM cell data analysis, doublets were excluded, and single cells were selected. Viability was assessed by flow cytometry using LIVE/DEAD TM Fixable Violet Dead Cell Stain Kit (Thermo Fisher Scientific, Waltham, MA, USA). Combining anti-CD38 and anti-CD138, plasma cells (CD38+CD138+), which include MM cells, were identified. The percentage of biotinylated isatuximab bound to plasma cells was identified by selecting streptavidin-APC positive cells (CD38+CD138+Streptavidin-APC+). The percentage of daratumumab bound to plasma cells was identified by selecting anti-IgG1-BV421 positive cells (CD38+CD138+ anti-IgG1-BV421+). The percentage of positive cells (% cells) and MFI of each individual marker was obtained using FlowJo version 10 (FlowJo LLC, Ashland, OR, USA). Data acquisition was performed on a MACSQuant Analyzer 10 flow cytometer (Miltenyi Biotec).

### 2.3. CD38 Recovery Assay

For the in vitro experiments evaluating the dynamics of CD38 expression, MM.1S cells (1 × 10^6^ cells in 5 mL tubes) were incubated with 1 µg/mL of daratumumab or unlabeled isatuximab, and the mean fluorescence intensity (MFI) of CD38 was measured at different time points (2, 4, 18, 26, 28, and 48 h). At 24 h of culture at 37 °C in a humidified incubator with 5% CO_2_ incubator, cells were washed at 300× *g* 5 min to remove daratumumab or unlabeled isatuximab from culture. After incubation, cells were stained with CD38-multi-epitope-FITC (Cytognos SL, Salamanca, Spain) for 15 min at RT. Subsequently, the cells were centrifuged at 300× *g* for 5 min and resuspended in 200 µL of PBS for acquisition by flow cytometry.

### 2.4. Viability Assay

MM cell lines (1 × 10^6^ cells/mL) were preincubated with increasing doses of isatuximab (0-, 0.1-, 0.01-, 1-µg/mL), 1 µg/mL of daratumumab or 10 µg/mL of isotype control (Immunotools, Friesoythe, Germany). Cells were analyzed after 72 h of culture at 37 °C in a humidified incubator with 5% CO_2_ incubator. Subsequently, cells were resuspended in Annexin-V Binding Buffer (10 mM HEPES, pH 7.4, containing 140 mM NaCl and 2.5 mM CaCl_2_) and stained with Annexin-V-PE (Miltenyi Biotec) for 15 min at RT in the dark. After washing with PBS, cells were resuspended with 200 µL of PBS, and propidium iodide (PI, Invitrogen, Eugene, OR, USA) was added. Data acquisition was performed on a MACSQuant Analyzer 10 flow cytometer (Miltenyi Biotec).

### 2.5. Migration and Adhesion Assays

For both the migration and adhesion assays, MM cell lines were fluorescently labeled with 10 μM of carboxyfluorescein-succinimidyl ester (CFSE; Sigma-Aldrich) for 10 min at 37 °C. Cells (1 × 10^6^ cells/mL) were preincubated for 30 min at 37 °C with increasing doses of isatuximab (0-, 0.01-, 0.1-, 1-µg/mL), 1 µg/mL of daratumumab, 10 µg/mL of isotype control, or relevant blocking antibodies: 25 µg/mL anti-CXCR4 (R&D Systems, Minneapolis, MN, USA) for migration assay or 25 µg/mL anti-CD49d (Thermo Fisher) for adhesion assay. Migration experiments was assessed using a transwell cell culture chamber (Corning, Corning, NY, USA). Then, preincubated cells were washed and added to the upper chamber, and 200 ng/mL of human CXCL12 (R&D Systems, MN, USA) resuspended in RPMI1640 containing 0.1% Bovine serum albumin (BSA; Roche Diagnostics, Manheim, Germany) was added to the lower chamber. After incubation for 6 h at 37 °C in a humidified incubator with 5% CO_2_ incubator, the number of cells that had migrated to the lower chamber was quantified. For adhesion assay, culture plates were coated with 10 µg/mL of vascular-cell adhesion molecule-1 (VCAM-1; R&D Systems) or 0.5% bovine serum albumin (BSA) as negative control overnight at 4 °C. The plates were washed with PBS, and preincubated cells were added to each well. After 1 h at 37 °C in a humidified incubator with 5% CO_2_, the wells were rinsed three times with PBS. Adhering fluorescently labeled MM cells were quantified. Fluorescence intensity, reflecting cell migration or adhesion, was measured using an Infinite M200 Pro Microplate reader (Tecan Group, Männedorf, Switzerland). The relative fluorescence units (RFUs) represent either the number of migrated or adhered cells, depending on the assay.

### 2.6. Statistics

Statistical analyses were performed using the GraphPad Prism 10 software. The normality of data distribution was assessed using the Kolmogorov–Smirnov test. Variables are presented as mean ± SEM or median (interquartile range [IQR]) depending on normal or non-normal distribution, respectively. Comparisons among three or more groups were tested by one-way analysis of variance (ANOVA) with Bonferroni post-hoc correction (parametric data) and the Friedman test with Dunn’s post-hoc correction (non-parametric data). For the CD38 recovery assay, within each time point (2, 4, 18, 26, 28, and 48 h), we compared three groups of cells: those treated with isatuximab, daratumumab, or an isotype control. For viability assays, within each antibody concentration of dose–response data (isatuximab or daratumumab at 0-, 0.01-, 0.1-, and 1-µg/mL), we compared three groups: antibody alone, antibody in combination with pomalidomide, and antibody in combination with lenalidomide. For migration and adhesion assays, dose–response data were compared to the isotype control condition. Correlation analyses were conducted using Spearman’s correlation coefficients. All *p*-values were based on a two-sided hypothesis, and those under 0.05 were considered statistically significant.

## 3. Results

### 3.1. Isatuximab and Daratumumab Do Not Compete for Binding to CD38 on MM Cell Lines

**Figure 1:**
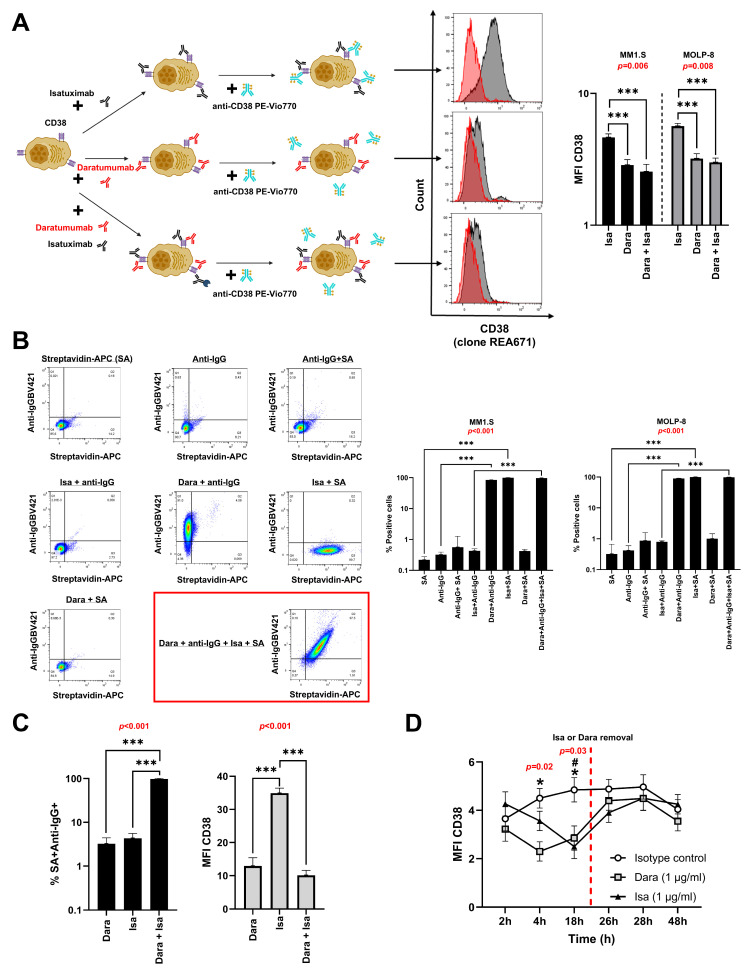
Differential binding of isatuximab (isa) and daratumumab (dara) to CD38 and their impact on antibody detection in MM cell lines. (**A**) Schematic representation of the non-competitive binding of isa and dara to CD38 (Created in BioRender. Osuna-Gómez, R. (2025) https://BioRender.com/ap6w4qo). Flow cytometry histograms show the expression of CD38 on MM cells treated with either isa, dara, or their combination. Bar graphs quantify the mean fluorescence intensity (MFI) of CD38 for each treatment (one-way ANOVA test with Bonferroni post-hoc). (**B**) Competitive binding assays using flow cytometry demonstrate the interaction of isa and dara in combination with anti-IgG1 and streptavidin (SA) staining. Panels show the effects of different combinations of antibodies (isa, dara, anti-IgG1, SA) in MM cells. Bar graphs summarize the percentage of SA and anti-IgG1 positive cells in both MM cell lines (Friedman test with Dunn’s post-hoc). (**C**) Plasma cells from BM of MM patients stained with isa and/or dara in combination with anti-IgG1 and SA. The bar graphs show the percentage of SA and anti-IgG1 positive cells (left) and the MFI of CD38 (right) on plasma cells (one-way ANOVA test with Bonferroni post-hoc). (**D**) MFI of CD38 on MM.1S cells after incubation with isotype control, dara, or isa at different time points (2, 4, 18, 26, 28, and 48 h). At 24 h, dara or isa were deleted (Friedman test with Dunn’s post-hoc). Comparison between conditions is indicated as follows: *: vs. dara; ^#^: vs. isa. Data represent mean ± SEM of five independent experiments or five different patients in (**C**). *p*-values from one-way ANOVA or Friedman test for each assay are shown in red. Statistical significance is indicated as follows: *, ^#^ *p* < 0.05, *** *p* < 0.001.

To evaluate by flow cytometry whether daratumumab and isatuximab compete for CD38 binding site, MM cell lines were labeled for 30 min with either daratumumab, biotinylated isatuximab, or a combination of both antibodies. As expected, biotinylated isatuximab specifically bound to streptavidin-APC, while daratumumab bound to anti-IgG1-BV421. After incubation, the cells were stained with anti-CD38-PE-Vio770 (clone REA671). Flow cytometry analysis was then used to measure CD38 expression. We found that biotinylated isatuximab does not compete with commercial clone REA671 for the CD38 binding site (Figure 1A). However, when cells stained with daratumumab or a combination of isatuximab plus daratumumab, they were negative for clone REA671, showing that daratumumab does compete with the clone REA671 for the binding site on CD38 (Figure 1A). The percentages of MM cells labeled separately with daratumumab or biotinylated isatuximab were comparable between groups (Figure 1B). Interestingly, when MM cells were labeled with both biotinylated isatuximab plus daratumumab, they bound to CD38 in a similar manner, suggesting that they do not compete for the CD38 binding site (Figure 1B).

### 3.2. Isatuximab and Daratumumab Do Not Compete for Binding of CD38 on BM Cells from MM Patients

Similarly, to the previously observation in MM cell lines, when BM plasma cells were labeled simultaneously with both biotinylated isatuximab and daratumumab, these antibodies did not compete for the CD38 binding site (Figure 1C). When BM plasma cells were treated with biotinylated isatuximab, the MFI of CD38 detected with clone REA671 did not decrease (Figure 1C). In contrast, when plasma cells were treated with daratumumab or with a combination of both antibodies, a significant decrease in the MFI was observed (Figure 1C). This occurs because, consistent with observations in MM cell lines, clone REA671 cannot detect CD38 in the presence of daratumumab.

### 3.3. CD38 Expression Rapidly Recovers on MM Cells Following Antibody Removal

To evaluate the dynamic recovery of CD38 expression on the surface of MM.1S cells after treatment with daratumumab or isatuximab, the cells were thoroughly washed to remove the antibodies, and recovery was analyzed at various time points. We determined CD38 levels using CD38-multi-epitope-FITC to avoid competition with the therapeutic antibodies. Incubation with the isotype control did not affect CD38 expression during the 48-h follow-up period (24 h after washing) (Figure 1D). Both daratumumab and isatuximab reduced CD38 expression, with the lowest levels observed at 18 h after antibody addition (Figure 1D). Interestingly, 2 h after washing (26 h post-initiation of the culture), CD38 expression on the cell surface was recovered to baseline levels, indicating a similar dynamic regulation of CD38 following antibody-mediated depletion/internalization (Figure 1D).

### 3.4. Pomalidomide Increases CD38 Levels and Enhance Isatuximab Apoptosis in MM Cell Lines

**Figure 2:**
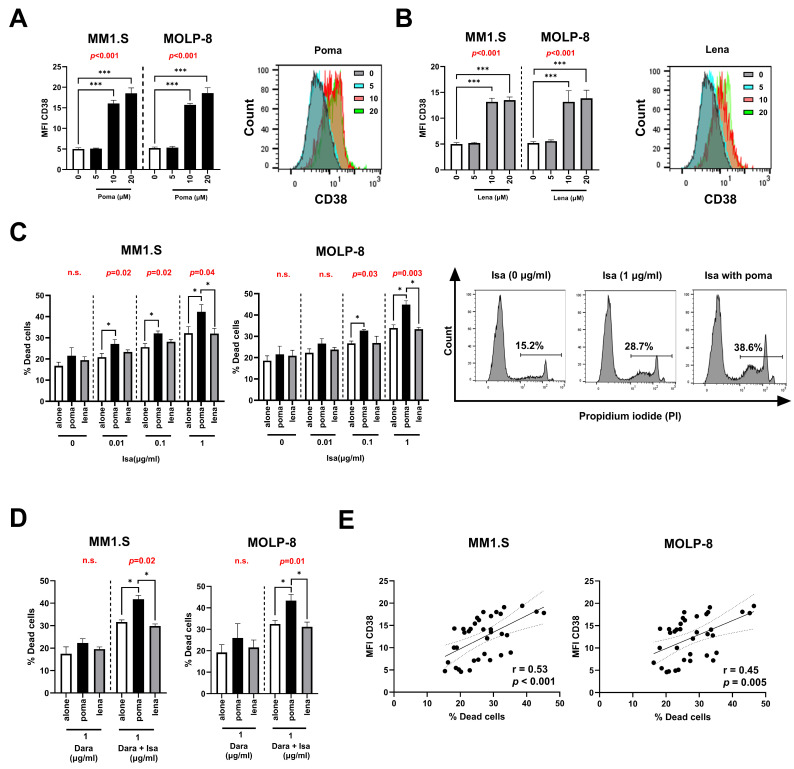
Pomalidomide and lenalidomide enhance CD38 expression and potentiate isatuximab-induced apoptosis in MM cell lines. Bar graphs show the mean fluorescence intensity (MFI) of CD38 on MM cell lines treated with increasing concentrations of (**A**) pomalidomide (poma) or (**B**) lenalidomide (lena) (0, 10, and 20 µM) (one-way ANOVA test with Bonferroni post-hoc). Flow cytometry histograms depict representative shifts in CD38 expression after treatment, indicating a dose-dependent increase. (**C**) Percentage of dead cells in MM cell lines treated with isatuximab (isa) (0, 0.01, 0.1, and 1 µg/mL) alone or in combination with poma or lena (left) (one-way ANOVA test with Bonferroni post-hoc and Friedman test with Dunn’s post-hoc). Representative flow cytometry histograms of propidium iodide (PI) staining illustrate the percentage of dead cells under different conditions (right). (**D**) Percentage of dead cells in MM cell lines treated with daratumumab (dara) or in combination with isa, either alone or in the presence of poma or lena (one-way ANOVA test with Bonferroni post-hoc and Friedman test with Dunn’s post-hoc). (**E**) Correlation analysis between MFI of CD38 and the percentage of dead cells in MM cells (Spearman-ranked correlation). Data are presented as mean ± SEM of five independent experiments. *p*-values from one-way ANOVA or Friedman test for each assay are shown in red (n.s. is not significant). Statistical significance is indicated as follows: * *p* < 0.05, *** *p* < 0.001.

We found that pomalidomide and lenalidomide significantly upregulated MFI of CD38 on MM cells when added at 10 µM or higher (Figure 2A,B). To explore the effect of IMiDs on isatuximab-induced apoptosis, MM cells were treated with isatuximab, either alone or in combination with 10 μM pomalidomide or lenalidomide, followed by annexin V/PI staining to assess cell viability. Isatuximab induced direct apoptosis in MM cells when added at 1 μg/mL. Pomalidomide, more potently than lenalidomide, increased the direct toxicity against MM cells induced by isatuximab (0.1 μg/mL or higher) (Figure 2C). However, this increase was not observed when MM cells were treated with isatuximab in combination with lenalidomide (Figure 2C).

On the other hand, when daratumumab was added alone or in combination with pomalidomide or lenalidomide, it did not induce direct apoptosis (Figure 2D). However, if under these conditions we added isatuximab, an increase in the apoptosis of MM cells was observed, being more potent in the presence of pomalidomide but not lenalidomide (Figure 2D). Further, we observed a positive correlation between the MFI of CD38 and the percentage of dead cells in MM cells (Figure 2E).

### 3.5. Isatuximab Interferes with In Vitro Migration and Cell Adhesion of MM Cells

**Figure 3:**
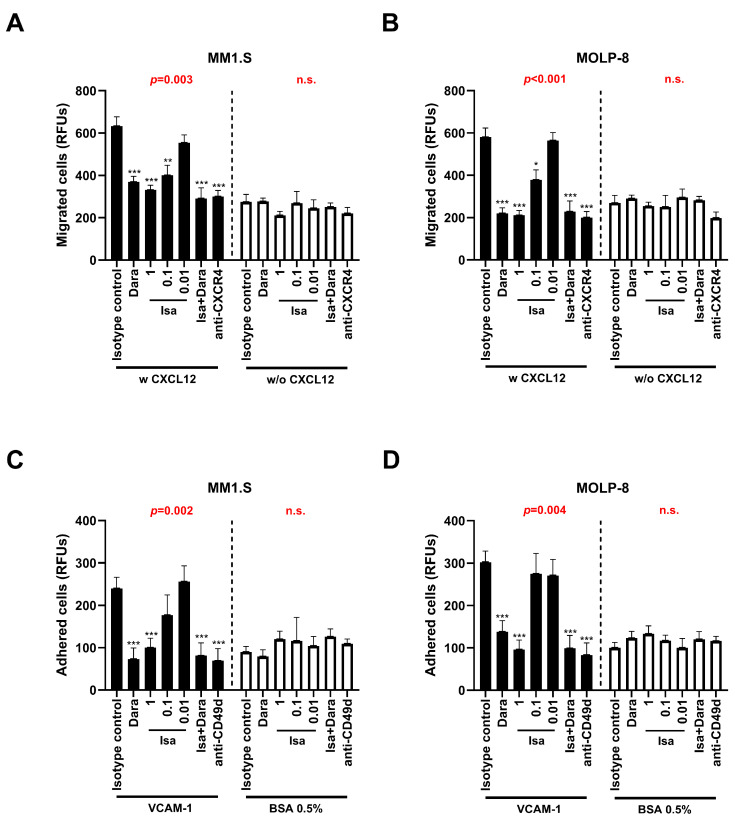
Inhibition of migration and adhesion by isatuximab (isa) and daratumumab (dara) in MM cell lines. (**A**,**B**) Migration assays were performed in the presence (w) or absence (w/o) of CXCL12 with isa and dara, either alone or in combination (one-way ANOVA test with Bonferroni post-hoc). The data are represented as relative fluorescence units (RFUs). (**C**,**D**) Adhesion assays were performed on plates coated with VCAM-1 or 0.5% BSA (control) in the presence of isa and dara, either alone or in combination (one-way ANOVA test with Bonferroni post-hoc). The data are represented as RFUs. Data are presented as mean ± SEM of five independent experiments. *p*-values from one-way ANOVA for each assay are shown in red (n.s. is not significant). Statistical significance is indicated as follows: * *p* < 0.05, ** *p* < 0.01, *** *p* < 0.001 compared to isotype control.

CD38 is known to synergize with the CXCR4 signaling pathway, influencing the chemotaxis and homing of MM cells. We evaluated the effect of isatuximab and daratumumab on MM cell migration using a CXCL12 gradient. An anti-CXCR4 antibody was used as a control of migration blockade. We found that daratumumab, isatuximab (0.1 μg/mL or higher) or the combination of both inhibited CXCL12-mediated migration in two MM cell lines when compared to isotype control, and they were comparable to anti-CXCR4 positive control (Figure 3A,B).

Additionally, we analyzed the effect of isatuximab and daratumumab on the CD49d/CD29-mediated adhesion of MM cells to VCAM-1, an essential component of the extracellular matrix. As shown in Figure 3C,D, daratumumab, isatuximab (1 μg/mL) or the combination of both significantly impeded the adhesion to VCAM-1 of MM cells, with no significant differences with anti-CD49d positive control.

## 4. Discussion

This study provides direct evidence that isatuximab and daratumumab bind to distinct, non-overlapping epitopes on CD38 and therefore do not compete for target engagement. This finding has important clinical implications, as it supports the possibility of sequential or even combinatorial use of these antibodies in MM treatment strategies. In the context of relapse after daratumumab, the lack of competitive binding suggests that isatuximab may still effectively engage CD38 and mediate antitumor activity. Furthermore, we show that treatment with IMiDs, particularly pomalidomide, upregulates CD38 expression and potentiates isatuximab-induced apoptosis, offering a potential strategy to enhance therapeutic efficacy. In addition, both antibodies were able to inhibit the migration and adhesion of MM cells, suggesting beneficial effects on tumor–microenvironment interactions beyond direct cytotoxicity. The distinct binding patterns observed by flow cytometry, together with previous structural studies [18,33], confirm that the epitopes recognized by isatuximab and daratumumab are different. Clinically, this non-competitive binding may help preserve CD38 accessibility after prior exposure to one antibody, potentially reducing cross-resistance and supporting their rational use in sequential or combination regimens [34].

Our results demonstrate the dynamic regulation of CD38 expression on MM.1S cells following daratumumab or isatuximab treatment and its subsequent recovery after washout. Both therapeutic antibodies significantly reduced CD38 expression, with the most pronounced depletion observed at 18 h post-incubation. This finding aligns with previous studies that highlight the rapid and effective downregulation of CD38 upon binding with daratumumab in MM cell lines [35]. Interestingly, we observed a rapid recovery of CD38 surface expression 2 h after the removal of either antibody, with CD38 levels returning to baseline at 26 h post-initiation of the culture. This suggests that MM cells possess mechanisms to rapidly replenish CD38 on their surface after antibody-mediated depletion. The rapid recovery of CD38 expression within two hours highlights the dynamic regulation of this antigen on MM cells. Importantly, this process is likely influenced by signals from the BM microenvironment, which may modulate CD38 availability in vivo. These findings suggest that CD38 is not irreversibly lost after antibody-mediated depletion but can rapidly reappear, a feature with potential implications for re-treatment and sequential strategies with anti-CD38 antibodies in relapsed disease. However, our in vitro experiment did not determine the reduction of concentrations of either antibody required for the recovery of CD38 expression. Further investigations are warranted to explore the underlying mechanisms of CD38 recovery and how they might be targeted to improve patient outcomes.

Furthermore, unlike daratumumab, isatuximab does not interfere with the detection of CD38 by clone REA671, both in MM cell lines and BM samples from MM patients. This property could facilitate the precise monitoring of the isatuximab response, with a robust tool for the detection of CD38+ MM cells. However, this effect may depend on the specific clone used for the detection of CD38. In a previous report, daratumumab was shown to interfere with CD38 detection via flow cytometry, depending on the detection clone [36].

We also demonstrated that pomalidomide and lenalidomide treatment significantly upregulates CD38 expression. This finding is consistent with previous research [37], which shows that IMiDs modulate immune responses and increase CD38 expression. Higher CD38 levels, as induced by IMiDs, provide more binding sites for isatuximab, amplifying its therapeutic efficacy. At the molecular level, this differential effect may be explained by the higher potency of pomalidomide compared with lenalidomide (approximately 10-fold stronger) and by its retained activity in lenalidomide-resistant cells, which together enhance its capacity to potentiate anti-CD38 antibody efficacy [38,39].

Our analysis of cytotoxicity revealed that isatuximab induces direct apoptotic effects at concentrations of 1 μg/mL, whereas daratumumab does not exhibit such effects. Moreover, clinical evidence indicates that isatuximab combined with pomalidomide and dexamethasone provides a significant overall survival benefit versus daratumumab with pomalidomide and dexamethasone, with favorable trends for progression-free survival and overall response rate [40,41]. Taken together, these results suggest that the improved clinical outcomes with isatuximab may be at least partly attributable to the direct apoptotic effect of isatuximab in MM cells.

Interestingly, isatuximab apoptosis, but not daratumumab, was significantly enhanced in the presence of pomalidomide, whereas lenalidomide did not produce a similar effect. These results align with preclinical data demonstrating that isatuximab and pomalidomide act synergistically to induce MM cell death [22]. In previous in vitro experiments, the pretreatment of peripheral blood mononuclear cells with 2 µM pomalidomide increased isatuximab-induced ADCC against MM cells [30]. This suggests that the upregulation of CD38 by pomalidomide enhances isatuximab apoptosis and may be responsible for this amplification.

In addition to apoptosis assay, both isatuximab and daratumumab inhibited MM cell migration and adhesion. The inhibition of CXCL12-mediated migration suggests that CD38 plays a critical role in the chemotactic response of MM cells, likely through synergy with the CXCR4 pathway. This finding is consistent with previous studies that underscore the role of daratumumab in MM cell migration and adhesion in MM cell lines [15]. Moreover, the inhibition of MM cell adhesion to VCAM-1 by both antibodies suggests that targeting CD38 can disrupt crucial interactions for MM cell retention in the bone marrow, potentially reducing drug resistance and improving treatment outcomes.

Collectively, the described mechanistic insights may contribute to improved resistance management, more rational sequencing of CD38-targeted antibodies, and better patient stratification in relapsed or refractory disease. First, the demonstration of non-competitive binding between isatuximab and daratumumab supports the rationale for the sequential or combinatorial use of these agents, particularly in patients who develop RRMM. Second, we observed that CD38 expression recovers after antibody removal. Although preliminary, this suggests that monitoring these agents could help optimize the timing of retreatment with anti-CD38 antibodies. Third, the ability of isatuximab to induce direct apoptosis, unlike daratumumab, represents a key mechanistic distinction that may contribute to the improved clinical outcomes reported with isatuximab-based regimens. Finally, we confirm that pomalidomide upregulates CD38 expression and thereby potentiates isatuximab-mediated apoptosis, providing a rationale for prioritizing this IMiD in combination strategies.

Despite the contribution of these results to our understanding the mechanisms of isatuximab, our study has some limitations. First, the in vitro nature of our experiments may not fully capture the complexity of the in vivo MM microenvironment, where interactions with the stroma and the immune system play pivotal roles. Second, our study focused on specific MM cell lines and bone marrow samples, which may not fully reflect the heterogeneity of MM patients. Further studies involving a larger patient cohort at different stages of disease are needed to confirm our findings. Lastly, while we explored the interactions of isatuximab and daratumumab in combination with IMiDs, the potential effects of other therapeutic agents and combinations warrant further investigation.

## 5. Conclusions

In conclusion, this study demonstrates that isatuximab and daratumumab do not compete for the same CD38 binding site, opening the door for potential sequential therapeutic administration in MM treatment. The unique binding characteristics of isatuximab, coupled with the modulatory effects of IMiDs on CD38 expression, underscore its promise in innovative treatment strategies for MM.

## Data Availability

All data relevant to the study are included in the article. The datasets used and analyzed during the current study are included in the article. They are available from the corresponding author on reasonable request.

## Abbreviations

The following abbreviations are used in this manuscript:

MM	Multiple myeloma
IMiDs	Immunomodulatory drugs
PIs	Proteasome inhibitors
RRMM	Relapsed/refractory MM
BM	Bone marrow
MFI	Mean fluorescence intensity
RT	Room temperature
RBC	Red blood cell
PI	Propidium iodide
CFSE	Carboxyfluorescein-succinimidyl ester
VLA-4	Very late antigen-4
VCAM-1	Vascular-cell adhesion molecule-1
Treg	Regulatory T cell
BSA	Bovine serum albumin
RFUs	Relative fluorescence units
IQR	Interquartile range
ANOVA	Analysis of variance
